# Antigen-specific T cell responses correlate with decreased occurrence of acute GVHD in a multicenter contemporary cohort

**DOI:** 10.1038/s41409-021-01456-x

**Published:** 2021-10-28

**Authors:** Conrad Russell Y. Cruz, Na Bo, Giorgos Bakoyannis, Kaylor E. Wright, Elizabeth A. Chorvinsky, Allison Powell, Catherine M. Bollard, David Jacobsohn, Kenneth R. Cooke, Christine Duncan, Robert M. Krance, Paul A. Carpenter, Courtney M. Rowan, Sophie Paczesny

**Affiliations:** 1 Center for Cancer and Immunology Research, Children’s National Medical Center, Washington, DC USA; 2 GW Cancer Center, George Washington University, Washington, DC USA; 3 School of Medicine, Indiana University, Indianapolis, IN USA; 4 Fairbanks School of Public Health, Indiana University, Indianapolis, IN USA; 5 Department of Oncology, Johns Hopkins Sidney Kimmel Comprehensive Cancer Center, Baltimore, MD USA; 6 Boston Children’s Hospital, Boston, MA USA; 7 Bone Marrow Transplant Division, Texas Children’s Hospital, Houston, TX USA; 8 Clinical Research Division, Fred Hutchinson Cancer Center, Seattle, WA USA; 9 Department of Microbiology and Immunology, Medical University of South Carolina, Charleston, SC USA

**Keywords:** Immunology, Adaptive immunity

## To the Editor:

Successful use of hematopoietic stem cell transplantation (HSCT) as a platform for pediatric immunotherapies is tempered by acute graft-versus-host disease (aGVHD). Several biomarkers that may distinguish beneficial from harmful immune responses have been validated in large cohorts [1, 2]. However, children comprised less than 10% of the subjects included in most of these studies. Although antigen-specific immune responses have been hypothesized to be impacted by cytokine storms, immune suppression from drugs, and graft-versus-host disease (aGVHD) [3], its correlation with aGVHD has not been formally established. Using data from a large cohort of pediatric HSCT recipients, we tested the hypothesis that post-transplant antigen-specific immune responses (IRs) negatively correlate with aGVHD.

Between 2013 and 2018, we enrolled 415 patients (286 children ≤ 18 years and 129 adults) across six centers: Children’s National Medical Center, Texas Children’s Hospital, Fred Hutchinson Cancer Research Center, Boston Children’s/Dana Farber cancer Institute, Johns Hopkins, and Indiana University on study NCT02194439 [4]. We subsequently screened antigen-specific IRs in peripheral blood mononuclear cells (PBMCs) taken from 53 evaluable patients (Supplementary Table 1). At a single selected time point post-transplant, and before the onset of GVHD, plasma biomarkers and antigen-specific immune responses were evaluated (see Supplementary Table 2). We selected patient timepoints where the collected samples occurred before or at the onset of GVHD.

Responses to viral (CMV, EBV, adenovirus, HPV, BK, flu, RSV, HHV6), and tumor (PRAME, SURVIVIN, WT1, MAGEA3) antigens were detected by IFN-γ ELISPOT. Antigen-specific cell IRs were defined as >50% greater responses to any stimuli when compared with the negative control (cells alone or actin) and at least 10 SFU/100,000 cells. These viral and tumor antigen-specific IRs were then combined into one variable: coded as 1 for a positive response and as 0 for a negative (or unevaluable) response. Immune responses occurring after aGVHD were not included in the analysis. For repeated measures in samples from the same patient, the earliest day on or after day 21 was chosen.

Biomarkers (ST2, IL-6, REG3a, TNFR1) were included among the response values. Chi-square tests to compare aGVHD ≥ 2 and the immune response variable as well as logistic regression between aGVHD ≥ 2 versus the variables immune response and biomarkers (ST2, IL-6, REG3a, TNFR1) [5–9] were performed. Age was also included as a covariate to account for its potential confounding effect. Odds Ratios (OR) with 95% confidence interval (95% CI) were summarized. *P* value less than 0.05 was considered as statistically significant.

**Fig. 1:**
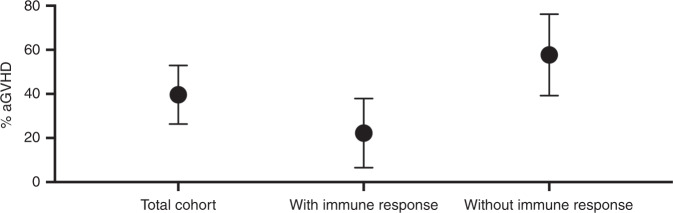
% GVHD present in the total cohort, in patients with immune responses, and in patients without immune responses. Confidence intervals are indicated by the error bars.

Twenty seven (50.9%) patients had IRs versus twenty six (49.1%) who did not. All IRs comprised responses against viral antigens; three IRs were responses against tumor antigens. To account for the potential confounding effect of age, we included it as a covariate. There is a significant inverse association between AGVHD and IR (OR = 0.1799, CI = 0.04177–0.6669, *p* = 0.0138) (Fig. 1). The odds of an AGVHD > 2 is higher when there is no detectable immune response (Table 1).

**Table 1:** Odds ratio of aGVHD as assessed by immune response and age and biomarker level (ST2, IL-6, REG3a, TNFR1).

Variable	OR	95% CI	*P* value
Immune response	0.1799	0.04177–0.6669	0.0138
Age in years	1.036	0.9978–1.081	0.0785
ST2	5.068	1.150–26.71	0.0394
IL6	1.459	0.3088–6.861	0.6266
Reg3a	1.951	0.4433–9.203	0.3792
TNFR1	0.2432	0.04384–1.082	0.0788

We also studied previously identified plasma biomarkers: ST2, IL-6, REG3a, TNFR1 [5–9]. We found that high ST2 levels in plasma were correlated with aGVHD, as we have previously observed (Table 1) [6]. In this subanalysis, IL-6, REG3a, and TNFR1 levels were not significantly associated with aGVHD ≥ 2.

To our knowledge, this is the first time that antigen-specific IRs have been demonstrated to be negatively associated with the subsequent development of aGVHD, and are also associated with high ST2 levels. ST2 is the most validated biomarker of aGVHD and death; [1, 6] we show similar findings and demonstrate that ST2 remains significantly associated with aGVHD as has been previously published [1, 4], while other biomarkers are not. Our findings have important implications for understanding the biology of aGVHD. Harmful graft versus host responses are often intertwined with beneficial (e.g., graft versus tumor) antigen-specific IRs [10]. Our findings suggest that these two processes may be uncoupled [11]. In theory, an alternative route for characterizing the risk for GVHD would involve monitoring antigen-specific immune reconstitution.

It is important to emphasize that T cell assays were completed on samples collected prior to the development of aGVHD, therefore ruling out immune suppression inherent to aGVHD or drugs used to treat it as the cause for the lack of immune response. We were unable to couple immune response with absolute lymphocyte counts (ALC), CMV serostatus, naïve/memory phenotype, and regulatory T cell function as these data were unavailable to us. However, in a previous publication (mostly in an adult cohort), we saw that ALC does not correlate with the presence of GVHD or with its biomarkers [12].

We recommend checking IR/GVHD biomarkers at post-transplant day 21, which is the earliest time point that was seen among patients with immune responses that can be linked to the presence or absence of GVHD.

In summary, these data suggest that functional antigen-specific T-cell profiles may favorably modulate the balance of donor-derived, allogeneic, T cell responses evolving early following HSCT. However, it has to be acknowledged that statistical association does not imply causation. Monitoring antigen-specific T-cell function and ST2 early post-HSCT (recommended day = 21) may facilitate the identification of patients at high risk for aGVHD which has important consequences in terms of preventive care.

## Supplementary information

Supplemental Material
