# Supplementary material for: Antigen-specific T cell responses correlate with decreased occurrence of acute GVHD in a multicenter contemporary cohort
Source: Bone Marrow Transplant. 2021 Oct 28;57(2):279–81. doi: 10.1038/s41409-021-01456-x (PMC8821012; doi:10.1038/s41409-021-01456-x)
Supplement: Supplementary file 1 — Supplemental Material [file 41409_2021_1456_MOESM1_ESM.pdf]

Supplementary Table 1: Additional patient information stratified by immune response*

	Entire Cohort n=53	+ Immune Response n=27	- Immune Response n=26	*p*
**Age (years)****	14.0 (IQR: 7, 43.5)	14 (IQR: 6, 33)	15 (IQR: 8, 44)	0.608
**Gender**				0.340
**Male**	32 (60.4%)	18 (56.2%)	14 (43.8%)	
**Female**	21 (39.6%)	9 (42.9%)	12 (57.1%)	
**Diagnosis**				0.215
**Malignant**	41 (77.4%)	19 (46.3%)	22 (53.7%)	
**Nonmalignant**	12 (22.6%)	8 (66.7%)	4 (33.3%)	
**Conditioning**				0.478
**Myeloablative**	42 (79.2%)	22 (52.4%)	20 (47.6%)	
**Reduced**	1 (1.9%)	1 (100.0%)	0 (0.0%)	
**Nonmyeloablative**	10 (18.9%)	4 (40.0%)	6 (60.0%)	
**Cell Source**				0.858
**Marrow**	24 (45.3%)	12 (50.0)	12 (50.0%)	
**Cord**	7 (13.2%)	3 (42.9%)	4 (57.1%)	
**PBSC**	22 (41.5%)	12 (54.5%)	10 (45.5%)	
**GVHD prophylaxis**				0.371
**CNI+MMF**	18 (34.0%)	9 (50.0%)	9 (50.0%)	
**CNI+MTX**	26 (47.2%)	11 (40.0%)	15 (60.0%)	
**CNI+MTX+ATG**	6 (11.3%)	5 (83.3%)	1 (16.7%)	
**CNI+PTCY**	1 (1.9%)	1 (100.0%)	0 (0.0%)	
**CNI+Sirolimus**	2 (3.8%)	1 (50.0%)	1 (50.0%)	
**aGVHD >/=2**	21 (39.6%)	6 (28.6%)	15 (71.4%)	0.008

* chi square analysis

** t test for age

Supplementary Table 2. Raw Coding of Immune Responses for Evaluated Samples.

Donor	day of biomarker	CMV	EBV	Adv	HPV	BK	Flu	HHV6	RSV	PRAME	Sur-vivin	WT1	MAGE-A3	AGVHD > 2	onset AGVHD or CGVHD 2-3	Age in years	Immune response	ST2	IL6	Reg3a	TNFR1	Condition-ning Prep	Cell source	GVHD prophy
CN-HSCT.022.D56	56	0	1	1	0	0	0	0	0	0	0	0	0	0	NA	2	1	11	2	28	1669	Full	Marrow	CNI + MTX + ATG
CN-HSCT.026.D61	61	1	0	0	0	0	1	0	0	0	0	0	0	0	NA	24	1	34	0	13	2250	Full	Marrow	CNI + MTX + ATG
CN-HSCT.028.D28	28	1	0	0	0	1	0	1	0	0	0	0	0	0	NA	18	1	25	180	17	6158	Full	Marrow	CNI + MTX
CN-HSCT.12.D56	56	1	1	0	0	0	0	0	0	0	0	0	1	0	NA	7	1	9	19	26	2826	Full	Marrow	CNI + MTX + ATG
CN-HSCT.20.D56	56	1	1	0	0	0	0	0	0	0	0	0	1	0	NA	6	1	12	2	36	3455	Full	Marrow	CNI + MTX + ATG
FH.HSCT.054.onsetGVHD (day 21)	21	0												1	21	13	0	121	99	17	3087	Full	PBSC	CNI + MTX + ATG
FH-HSCT.002.UNSC1 (D49)	49	0	0											1	49	52	0	146	37	28	11331	Full	PBSC	CNI + MTX
FH-HSCT.005.D100	100	0	0	0	0									0	NA	53	0	10	13	81	11548	Full	PBSC	CNI + MTX
FH-HSCT.027.D28	28	0	0	0	0	0	0	0	0	0	0	0	0	0	NA	42	0	19	61	26	15281	NonMyeloablative	PBSC	CNI + MMF
FH-HSCT.035.D56	56	1	1	0	0	0	0	0	0	0	0	0	0	0	NA	61	1	21	6	87	10434	NonMyeloablative	PBSC	CNI + MMF
FH-HSCT.038.D28	28	0	0	0	0	0	0	0	0	0	0	0	0	1	83	61	0	4	2	31	6059	Full	PBSC	CNI + MTX
FH-HSCT.039.D56	56	1	0	0	0	0	0	0	0	0	0	0	0	0	NA	49	1	9	5	51	5721	Full	PBSC	CNI + MTX
FH-HSCT.040.D28	28	0	1	0	0	0	0	0	0	0	0	0	0	1	50	33	1	10	6	30	2479	Full	PBSC	CNI + MTX
FH-HSCT.043.D28	28	0	0	0	0	0	0	0	0	0	0	0	0	1	36	61	0	45	20	54	9872	NonMyeloablative	Marrow	CNI + MMF
FH-HSCT.044.D28	28	0	0	0	0	0	0	0	0	0	0	0	0	1	52	44	0	5	8	22	3667	NonMyeloablative	PBSC	CNI + MMF
FH-HSCT.053.Onset GVHD (D28)	28	0	0	0										1	28	13	0	65	7	13	2163	Full	Cord	CNI + MMF
FH-HSCT.064.Onset GVHD (Day 21)	21	0	0											1	21	8	0	106	141	157	3027	Full	Cord	CNI + MMF
FH-HSCT.072.D28	28	0	0	0	0	0	0	0	0	0	0	0	0	1	33	12	0	79	96	124	2834	Full	Cord	CNI + MMF
I.HSCT.177.D21	21	1	0	0	0	0	0	0	0	0	0	0	0	1	42	8	1	7	2	13	604	Full	Cord	CNI + MMF
I-HSCT.003.d270	270	1	0	0	0	0				0				0	NA	3	1	11	11	33	2586	Full	Cord	CNI + MMF
I-HSCT.020.D100	100	0	0	0	0	0	0	0	0	0	0	0	0	0	NA	20	0	21	0	284	3511	NonMyeloablative	Cord	CNI + MMF
I-HSCT.028.D60	60	1	0	0	0	1	0	0	0	0	0	0	0	0	NA	15	1	29	278	35	5440	Full	Marrow	CNI + MTX
I-HSCT.041.d30	30	1												1	34	4	1	22	73	18	2319	Full	Marrow	CNI + MTX
I-HSCT.043.d150	150	1	1	0	0	0				0	0			0	NA	64	1	14	2	43	5505	NonMyeloablative	PBSC	CNI + MMF
I-HSCT.044.d30	30	1	0	0										0	NA	54	1	7	4	130	3174	NonMyeloablative	PBSC	CNI + MMF

I-HSCT.056.d30	30	0	0											1	57	59	0	143	24	53	6980	NonMyeloablative	PBSC	CNI + MMF
I-HSCT.059.D60	60	1	1	0	0									0	0	33	1	26	35	6	3535	Full	PBSC	CNI + sirolimus
I-HSCT.067.D30	30	0	0	0	0	0	0	0	0	0	0	0	0	0	NA	43	0	19	8	30	8185	Full	PBSC	CNI + sirolimus
I-HSCT.070.D21	21	0	0	0	0									1	22	65	0	244	84	1419	26047	NonMyeloablative	PBSC	CNI + MMF
I-HSCT.082.D21	21	1	1	0	0	0	1	0	0	0	0	0	0	1	128	45	1	18	7	23	3099	Reduced	PBSC	CNI + MMF
I-HSCT.084.D21	21	1	1	0	0	0				0				1	123	70	1	50	21	43	5793	NonMyeloablative	PBSC	CNI + MMF
I-hsct-016.D30	30	1	1	0										0	NA	13	1	124	205	2	6769	Full	PBSC	CNI + MTX
SK-HSCT.020.D177	177	1	0	0	0	0				0				0	NA	4	1	15	101	60	2034	Full	Marrow	CNI + MMF
TC.HSCT.001.D56	56	0	0	0	0	0				0				0	NA	8	0	16	23	18	1427	Full	Marrow	CNI + MTX
TC.HSCT.005.D13	13	1	1	1	1	0	0	0	1	0	0	0	0	0	NA	21	1	131	75	26	8987	Full	Marrow	CNI + MTX
TC.HSCT.006.D100	100	0	0	0	0	0								0	NA	4	0	303	55	385	13135	Full	Marrow	CNI + MTX
TC.HSCT.007.D028	28	1	0	0	0	0				0				0	NA	7	1	20	0	14	2468	Full	PBSC	CNI + MTX + Steroids
TC.HSCT.008.D56	56	0	0	0	0	0	0	0	0	0	0	0	0	0	NA	12	0	23	10	17	3440	Full	Marrow	CNI + MTX
TC.HSCT.010.D28	28	1	0	0	0	1	0	0	0	0	0	0	0	0	NA	14	1	19	52	47	2393	Full	PBSC	PTCy + CNI
TC.HSCT.013.D100	100	0	0	0	0									0	NA	4	0	12	48	22	2927	Full	Marrow	CNI + MTX
TC.HSCT.015.D28	28	0	0	0	0	0	0	0	0	0	0	0	0	1	54	3	0	11	10	15	3859	Full	Marrow	CNI + MTX
TC.HSCT.016.D100	100	1	0	0	0	0	0	0	0	0	0	0	0	0	NA	6	1	44	3	23	3786	Full	Marrow	CNI + MTX + ATG
TC.HSCT.017.D28	28	0	0	0	0	0	0	0	0	0	0	0	0	1	31	17	0	24	317	37	4085	Full	Marrow	CNI + MTX
TC.HSCT.018.D56	56	1	0	0	0	0	0	0	0	0	0	0	0	0	NA	20	1	33	16	26	4085	Full	Marrow	CNI + MTX
TC.HSCT.019.D100	100	1	0	1	0	0	0	0	0	0	0	0	0	1	166	10	1	116	48	49	9725	Full	PBSC	CNI + MTX
TC.HSCT.020.D56	56	0	0											0	NA	4	0	9	16	23	5753	Full	Marrow	CNI + MTX
TC.HSCT.023.D100	100	0	1	1	0	1				0				0	NA	7	1	7	8	20	1093	Full	Marrow	CNI + MTX
TC.HSCT.029.D56	56	0												0	NA	18	0	14	19	25	3682	Full	Marrow	CNI + MTX
TC.HSCT.036.D100	100	0	0											0	NA	1	0	21	35	28	1928	Full	Marrow	CNI + MTX
TC.HSCT.038.d56	56	0	0	0										1	286	12	0	18	93	50	1850	Full	Marrow	CNI + MTX
TC-HSCT.014.D180	180	0	0	0	0									1	24	22	0	17	366	35	7222	Full	PBSC	CNI + MTX
TC-HSCT.026.D100	100	1	1	0	0	1	0	0	0	0	0	0	0	0	NA	4	1	35	78	12	4421	Full	Cord	CNI + MMF
TC-HSCT.034.D28	28	0	0	0	0	0	0	0	0	0	0	0	0	1	81	8	0	46	11	9	2255	Full	Marrow	CNI + MTX
